# A nutrient-sensing pathway activated in APC-driven colorectal cancer

**Published:** 2014-01

Truncating mutations in the tumour suppressor gene encoding APC are the most common cause of colorectal cancer. APC is a negative regulator of the Wnt signalling pathway, and constitutive activation of Wnt–β-catenin signalling is thought to form the basis of the oncogenic effects of *APC* mutations. However, recent evidence has suggested that other proteins could also be involved. Alexander Valvezan, Peter Klein and colleagues recently showed that APC suppresses mTORC1 activity *in vitro*. Here, the group explored the relationship between APC and mTORC1, a nutrient sensor that regulates cell growth and proliferation, *in vivo*. The authors show that mTORC1 activity is markedly upregulated in *apc* mutant zebrafish and in intestinal polyps in *Apc* mutant mice. mTORC1 inhibition suppressed multiple developmental defects caused by *apc* mutation in zebrafish. Interestingly, combined inhibition of mTORC1 and Wnt signalling was needed to restore normal body curvature. These findings suggest that mTORC1 is a key mediator of *APC*-driven tumorigenesis and developmental defects, and suggest mechanistic overlap with other polyposis syndromes that are associated with mTORC1 activation. Page 63

